# Early Diagnosis of Huntington Disease: Insights from Magnetic Resonance Spectroscopy—A Systematic Review

**DOI:** 10.3390/jcm13216390

**Published:** 2024-10-25

**Authors:** Pablo S. Martínez Lozada, José Duque Perez, Ronney Celi Salinas, Bryan Miranda Morales, Juan Francisco Pazmiño Mesías, Cecilia Alejandra García Ríos, Jose A. Rodas, Jose E. Leon-Rojas

**Affiliations:** 1 NeurALL Research Group, Quito 170157, Ecuador; martinez.sebastian2503@gmail.com (P.S.M.L.); josedanielduque1@hotmail.com (J.D.P.); ronneycelis@hotmail.com (R.C.S.); miranda_bryan@hotmail.com (B.M.M.); juanfrapazm@gmail.com (J.F.P.M.); 2 Medical School, Universidad de las Américas (UDLA), Quito 170124, Ecuador; 3 Medignosis, Research Department, Quito 170157, Ecuador; 4 Christus Muguerza Hospital Alta Especialidad, Monterrey 64060, Mexico; 5 Facultad Ciencias de la Salud, Universidad Nacional de Chimborazo, Riobamba 060108, Ecuador; 6 School of Psychology, University College Dublin, D04 V1W8 Dublin, Ireland; 7 Escuela de Psicología, Universidad Espíritu Santo, Samborondón 092301, Ecuador

**Keywords:** Huntington disease, magnetic resonance spectroscopy, pre-symptomatic Huntington disease, neuroimaging

## Abstract

**Background/Objectives**: Huntington’s disease (HD) is a fully penetrant neurodegenerative disease with a profound effect on quality of life. In recent years, there has been rapid growth in the description of its pathogenesis and diagnosis. Magnetic resonance spectroscopy (MRS) measurements can aid in the discrimination between premanifest Huntington’s disease (Pre-HD) and healthy control (HC) subjects to establish early supportive and symptomatic management. Our objective was to evaluate metabolic changes using MRS to shed light on its potential as a biomarker through a systematic review. **Methods**: We followed the PRISMA guidelines, extracting articles from PubMed, Scopus, and the Virtual Health Library. We included patients with pre-HD, HD, and HC subjected to MRS, reporting the concentration of metabolites in at least one brain region. **Results**: In the putamen, N-acetyl Aspartate (NAA) was significantly decreased in 77.9% and total NAA (tNAA) was decreased in 72.4% of cases; no significant difference was found in 27.5% (*n* = 19) of cases. Furthermore, when looking into HD vs. pre-HD in the putamen, tNAA and NAA were decreased in 100% of participants. In the caudate nucleus, NAA and creatine were significantly decreased in 100% of HD in comparison to pre-HD participants, whereas tNAA showed a significant decrease in only 50%. **Conclusions**: MRS can be a relevant tool for the early diagnosis of HD; potential objective biomarkers related to its onset and pathogenesis exist and show differences between controls, pre-HD and HD patients. However, an effort should be made to standardize MRS methodology and reporting in subsequent studies.

## 1. Introduction

Huntington’s disease (HD) is an autosomal dominant disease caused by a trinucleotide repeat (CAG) in the huntingtin gene located on chromosome 4; the number of repetitions is directly related to the progression of the disease. Patients with more than 39 CAG repeats almost always develop signs and symptoms of the disease, and 36–39 repeats cause reduced penetrance [1]. This mutation causes disruption of proteostasis, transcription and mitochondrial function and direct toxicity to the mutant protein, resulting in neuronal loss and gliosis in the basal ganglia (mainly the putamen and caudate nucleus), whose primary function is coordinating movement [1].

HD encompasses a syndrome of movement, neuropsychiatric abnormalities, and progressive cognitive and behavioural impairment [1]. Symptom onset takes place around 40 years of age, and it is more common in males and people of Western European descent (prevalence of 10.6–13.7 per 100,000) [2]. In the early stages, the clinical manifestations can be subtle and nonspecific; affected individuals show worsening movement abnormalities (chorea, incoordination, dystonia, rigidity, motor persistence, and slowed saccadic eye movements), cognitive decline (in areas such as organization, planning, and speech), and neuropsychiatric manifestations (depression, suicidal ideation) [3]. Death usually occurs around 15–20 years from disease onset due to complications such as falls, inanition, or aspiration pneumonia [3].

Diagnosis of HD is generally achieved using a combination of the patient history (family history and clinical manifestations) and genetic testing (polymerase chain reaction) [2]. Imaging is rarely used to date, even though some imaging modalities can detect the disease up to 25 years before onset; these include structural magnetic resonance imaging (MRI), diffusion-weighted MRI, functional MRI and FDG PET scans [1,2,3]. Typical findings on imaging include atrophy of the basal ganglia (mainly of the caudate head) and ex vacuo ventriculomegaly [2]. Another technique, using MRI, can quantify metabolic changes in the brain by applying spectroscopic analysis of the MRI signal; this technique is known as magnetic resonance spectroscopy (MRS) and has also been used in HD patients.

MRS is a technique that identifies and quantifies the presence of different metabolites in different tissues; this quantification is possible because the distribution of electrons within an atom causes the nuclei of different molecules to experience a different magnetic field [4]. MRS was limited to the research field, but it is increasingly used in the clinical setting of different pathologies. Due to its sensitivity, small mobile molecules present in millimolar quantities can be detected. At field strengths such as 1.5 or 3.0 T, only choline, creatine, and N-acetyl aspartate signals are observed in the normal brain at long echo times (e.g., 140 or 280 ms); compounds such as lactate, alanine, or others can be detected in pathological conditions [4]. At short echo times (e.g., 35 ms or less), other compounds such as glutamate, glutamine, and myoinositol, as well as lipids and other macromolecules, can be detected [4]. Certainly, MRS has been utilized in various neurodegenerative conditions including Alzheimer’s disease, Parkinson’s disease, and Huntington’s disease [4,5]. In the context of Huntington’s disease, which involves a progressive degeneration of the basal ganglia, MRS is employed to measure the variations in metabolites as a biomarker in these specific brain regions [5]. Unlike MRI, which focuses on anatomical structures and the quantification of the volumes of specific structures (i.e., the caudate nucleus), MRS detects changes in specific metabolites such as N-acetyl aspartate, creatine, and choline [4,5]. Detection of these metabolites and their changes can show a metabolic alteration even before structural abnormalities appear, offering hope for early intervention and disease management [4,5]; this makes MRS particularly valuable in diagnosing and monitoring neurodegenerative diseases like HD.

Therefore, the purpose of our article is to explore the utility of MRS as a tool for the early diagnosis of HD. Our scope encompasses the potential of MRS in identifying objective biomarkers associated with the onset and progression of HD, as well as considering the implications of early diagnosis. We aim to identify the most common metabolic changes between healthy controls (HCs), pre-symptomatic Huntington disease (pre-HD) patients, and HD patients. However, to fully harness the clinical utility of MRS, it is essential to address methodological challenges and establish standardized protocols for data acquisition, processing, and reporting. Collaborative efforts towards methodological harmonization will facilitate the translation of MRS findings into clinical practise, ultimately benefiting individuals affected by neurodegenerative disorders, including HD.

## 2. Materials and Methods

This systematic review was registered (ID: CRD42024495834) in the International Prospective Register of Systematic Reviews (PROSPERO) and followed the guidelines of the Preferred Reporting Items for Systematic Reviews and Meta-Analysis (PRISMA).

### 2.1. Eligibility Criteria

We included articles from PubMed, Scopus, and the Virtual Health Library (VHL) from inception until 2 February 2024; case reports, case series, cross-sectional studies, cohort studies, and experimental studies were included. Only articles that included patients in the early stages of HD were considered; these patients had (1) a presence of symptoms and clinical signs evidenced by standardized scales such as the Shoulson and Fahn capacity scale and the Unified Huntington’s Disease Rating Scale (UHDRS); (2) a parent with proven HD; and/or (3) a genetic diagnosis showing at least 36 CAG repeats in the huntingtin gene on chromosome 4. Additionally, these articles had to report the use of nuclear magnetic resonance spectroscopy (MRS) and the concentration of individual metabolites in at least one specific brain region. These metabolites were needed to compare patients in the early stages of HD to patients with established HD and healthy controls (HCs). Included studies needed to always consider pre-HD patients and compare them with HC and/or HD patients. Exclusion criteria included studies that did not consider patients with early symptomatic HD (i.e., studies that considered only HD patients or HD patients compared with only HC patients) and patients with no known DNA diagnosis or no direct familial history of HD; we also excluded letters to the editor, commentaries, and narrative and systematic reviews.

### 2.2. Search Strategy

Medical subheadings (MeSHs) and text words related to Huntington’s disease, Huntington’s Corea, Huntington’s dementia, and nuclear magnetic resonance spectroscopy were used in our search strategy; no filters were used. PubMed (from the beginning of the database to 2 February 2024), VHL (from the beginning of the database to 2 February 2024), and Scopus (from the beginning of the database to 2 February 2024) were queried; the full search strategy can be found in the Supplementary File S1. Specific search strategies were developed under the guidance of NeurALL research group staff with expertise in systematic review searches. The search was conducted independently by two authors (J.D.P and P.M.L); when in disagreement, consensus was reached between both parties.

### 2.3. Data Management

The results of the literature search were imported and managed in Rayyan, a web application for systematic reviews developed by the Qatar Computing Research Institute (https://www.rayyan.ai/) (accessed on 2 February 2024). Rayyan mitigates data entry errors, avoids mistakes during the removal of duplicate articles and reduces the risk of bias during the selection and decision process.

### 2.4. Selection Process

Two independent authors, who were blinded to each other’s decisions, reviewed all the titles and abstracts according to the aforementioned eligibility criteria. Once reviewed, the full text of each included article was examined; any discrepancy was solved by mutual consensus and by the inclusion of a third reviewer. Articles that met the inclusion criteria were added to the systematic review.

### 2.5. Data Items

The data extracted from the selected articles were collected into a Microsoft Excel spreadsheet and included the following: author, DOI (Digital Object Identifier), type of study, number of participants, and their demographic information; they were classified into pre-HD, HD and HC. Technical data of MRS were also included, such as type of equipment, model, field strength (Tesla), sequence time, TR/TE, and the anatomical area being studied. Finally, the metabolites and their concentrations were also collected.

### 2.6. Data Synthesis

From each selected study, we extracted the total amount of the reported metabolites in every brain region; these data were later compared between diagnostic groups (pre-HD, HD and HC) by using relative and absolute frequencies. Meta-analysis was not feasible due to the heterogeneity of MRS techniques. To address the heterogeneity in MRS techniques, we compared metabolite concentrations across studies using a qualitative approach that focused on analyzing changes in metabolites, independent of MRS field strength and sequences, classifying them as increased, decreased or no change based on the reported statistical power of each study (i.e., *p*-value and confidence intervals); a significant change was considered when the study reported a *p*-value of <0.05.

### 2.7. Risk of Bias

Risk of bias was assessed by two independent reviewers, blinded to each other’s decision, using the National Heart, Lung, and Blood Institute (NHLBI) Study Quality Assessment Tools (https://www.nhlbi.nih.gov/health-topics/study-quality-assessment-tools) (accessed on 2 January 2024); any discrepancy was resolved by a third reviewer. Studies were classified as having a minimally low, moderately low, or high risk of bias. If a study received affirmative responses to 80% or more of the questions, it was classified as good, indicating a minimally low risk of bias; if it received affirmative responses to 50–79% of the questions, it was classified as fair, indicating a moderately low risk of bias; and if it received affirmative responses to less than 50% of the questions, it was classified as poor, indicating a high risk of bias.

## 3. Results

**Figure 1:**
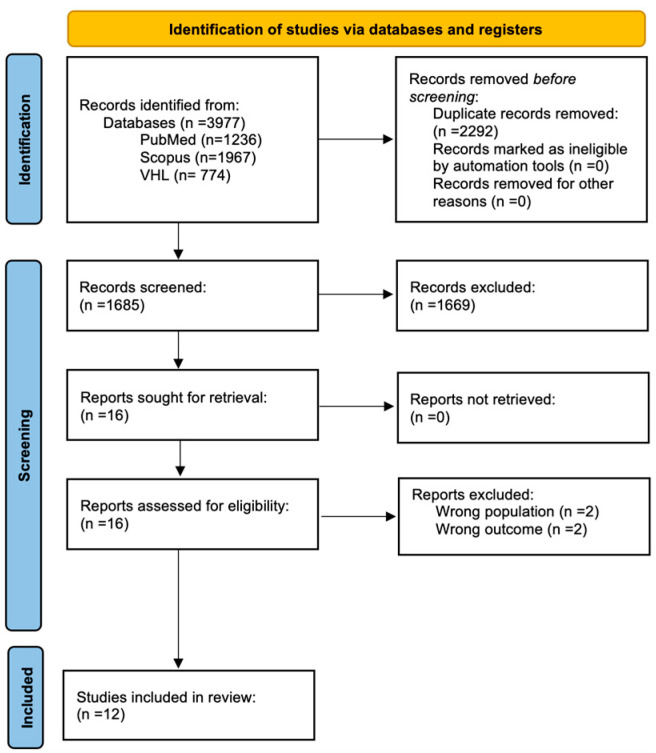
PRISMA 2020 flow diagram showcasing the selection process.

We identified 3977 potentially relevant studies in the PubMed, Scopus, and VHL databases; after removing duplicates and applying our eligibility criteria, we obtained a total of 12 articles for inclusion in our review [5,6,7,8,9,10,11,12,13,14,15,16]. The full selection process can be seen in Figure 1. These 12 studies included a total of 146 HC, 155 pre-HD, and 170 patients diagnosed with HD.

Bias assessment of the 12 included studies resulted in categorizing 66.6% of studies as having a moderately low risk of bias and 33.4% as having a high risk of bias (Table 1).

### 3.1. Magnetic Resonance Spectroscopy—Technical Characteristics

Most studies were performed using magnetic fields of 3 T (*n* = 5) [5,7,8,15,16] and 1.5 T (*n* = 4) [6,9,11,12], with two studies using 7 T [10,14] and one using 0.5 T [13]. The most used sequence was PRESS (*n* = 7) [7,8,9,11,13,15,16], and two studies used STEAM [10,11]; GRADIENT ECHO, PROBE, MPRAGE and SPARS sequences were used in one study each [5,6,12,14]. The TE ranged from 3.2 to 288 ms (mean: 60.54). The TR ranged from 7 to 3500 ms (mean: 1668.16). The variability in MRS results across studies in our analysis poses notable challenges for drawing definitive conclusions. Most studies utilized a magnetic field strength of 3 T (*n* = 5), which enhanced sensitivity and resolution compared to those using a strength of 1.5 T (*n* = 4). However, the diverse sequences employed, mainly PRESS (*n* = 7), had a profound impact on metabolite localization and quantification, introducing significant variability. Fluctuations in TE and TR further complicate comparisons, as these parameters influence metabolite visibility and consistency. These differences underscore the necessity for greater standardization in future MRS studies to improve the comparability and reliability of results.

### 3.2. Brain Metabolites

We found a high degree of heterogeneity between the reported results (this was also evident when assessing the technical characteristics of the MRS used in each study). Therefore, we decided to organize the results of our review according to the metabolite and the anatomical area being studied; the results were further sub-divided by comparing the results of pre-HD vs. HC, and HD vs. pre-HD. Due to variations in MRS methodology and acquisition, it was not feasible to provide an overall summary statistic for each metabolite, so we expressed the overall findings qualitatively as “no change”, “decrease”, and “increase”. The results of each metabolite are summarized in Table 2, Table 3, Table 4, Table 5, Table 6, Table 7, Table 8 and Table 9.

### 3.3. Changes Per Anatomical Area

**Figure 2:**
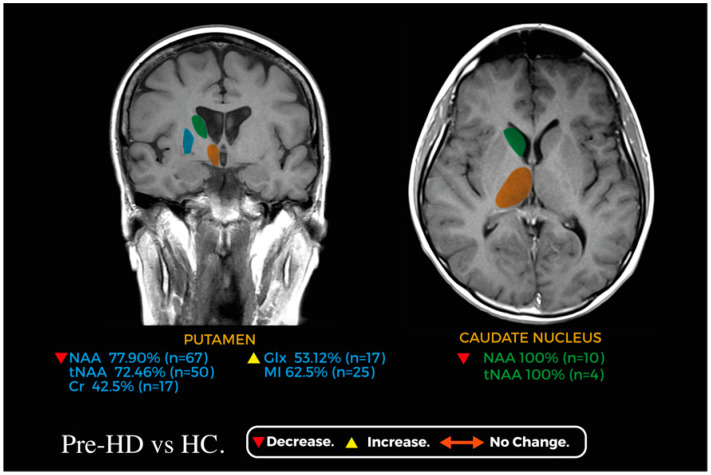
Coronal and axial planes of a magnetic resonance image representing the main changes in the metabolic profile of the caudate nucleus (green) and putamen (blue) between pre-HD and HC; the thalamus (orange) is shown for reference.

**Figure 3:**
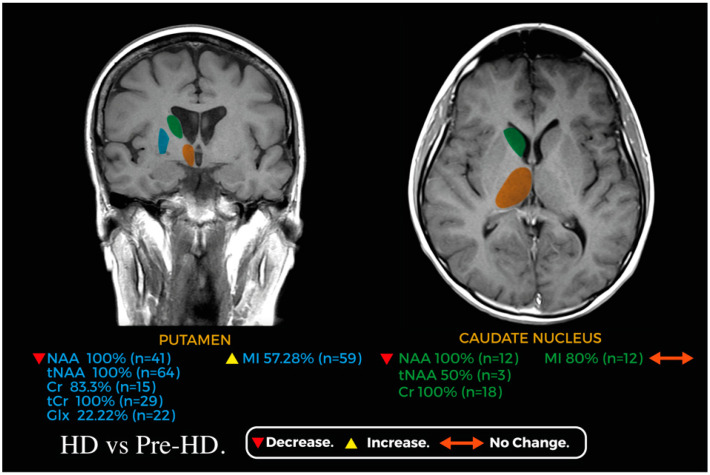
Coronal and axial planes of a magnetic resonance image representing the main changes in the metabolic profile of the caudate nucleus (green) and putamen (blue) between HD and pre-HD; the thalamus (orange) is shown for reference.

Figure 2 and Figure 3 showcase relevant metabolic changes between pre-HD vs. HC and pre-HD vs. HD, respectively.

#### 3.3.1. Frontal Cortex

We found a significant decrease in the concentration of N-acetyl-aspartate (NAA), glutamate (Glu), and choline (Cho) in pre-HD patients when compared with HC patients; this difference did not occur for creatine, glutamate/glutamine (Glx) and myoinositol (mI) [5]. Similarly, when looking into HD vs. pre-HD, the NAA/Cho ratio was significantly increased [6], but the Cho/Cr ratio, Cho, total NAA (tNAA), Cr, Glx, and mI showed no differences in concentration even at 24 months of follow-up [6,14]

#### 3.3.2. Occipital Cortex

We found no difference in the concentrations of tNAA and Cr between pre-HD and HC [7]; we also found no significant difference in the lactate (Lac)/NAA ratio [11]. In contrast, when looking into HD vs. HC, one study found an astounding difference in the Lac/NAA ratio with concentrations increasing up to 11.6 ± 4.1% in the HD subjects in comparison to 3.7 ± 1.5% in HC subjects (*p* < 10^−11^) [11]. In the same study, increases in the Cho/NAA (*p* < 0.01) and Cho/Cr ratios (*p* < 0.02) were reported between HD patients and HC; however, there was no difference in the NAA/Cr ratio between these two groups [11].

#### 3.3.3. Prefrontal Cortex and Hypothalamus

In the hypothalamus and prefrontal cortex, when comparing pre-HD vs. HC, only one study compared 12 HD and 14 pre-HD patients; no statistically significant difference was found for NAA, Cho, Cr, Lac, Glx, or mI between these two groups [10].

#### 3.3.4. Thalamus

In the thalamus, when comparing pre-HD vs. HC, only one study compared 19 pre-HD individuals to 8 HC individuals; no statistically significant difference was found for NAA, Cho, or Cr between the participants of this study [9]. In a similar fashion, when comparing 12 HD vs. 14 pre-HD participants, no statistically significant difference was found for NAA, Cho, Cr, Lac, Glx or mI [10].

#### 3.3.5. Striatum

In the striatum, when analyzing pre-HD vs. HC, NAA and Cr were significantly decreased in 33% and 100% of patients, respectively [11,12]. In contrast, Cho and Lac were significantly elevated in 66% and 100% of pre-HD patients, respectively [11]. However, when looking into HD vs. pre-HD patients, there was no difference in the reported Cho concentrations, but NAA and Cr were significantly decreased in HD patients [12].

#### 3.3.6. Putamen

**Figure 4:**
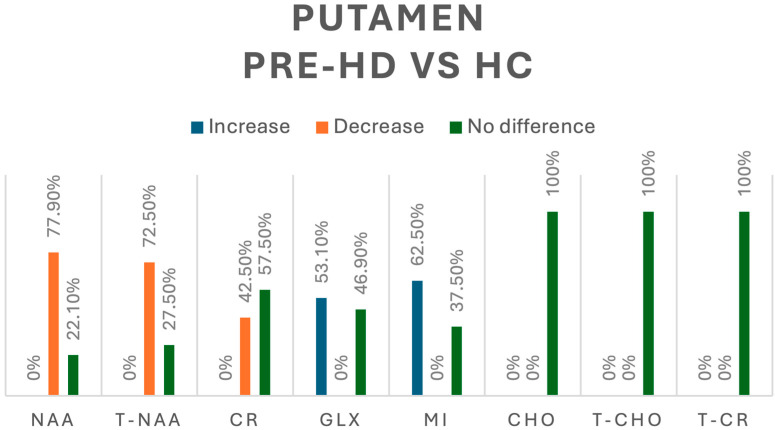
Comparison of metabolites in the putamen between pre-HD patients and healthy controls (HCs). N-acetyl-aspartate (NAA), total N-acetyl-aspartate (T-NAA), creatine (CR), glutamate/glutamine (GLX), myoinositol (MI), choline (CHO), total choline (T-CHO), and total creatine (T-CR).

In our review, when looking into the differences between pre-HD patients and healthy controls (HCs), NAA was assessed in 86 pre-HD patients [8,9,13,15]—we found a decrease in its concentration in 77.9% and no significant difference in 22.1% of cases. When looking into t-NAA in 69 pre-HD patients [8,15,16], we found an overall decrease in 72.5% and no significant difference in 27.5% of cases, whereas Cr was assessed in 40 pre-HD patients [8,9,13] with a decrease in its concentration in 42.5% and no significant difference in 57.5% of cases. Regarding Glx, assessed in 32 pre-HD patients [13,16], we found increases in 53.1% and no difference in 46.9% of cases. Myoinositol (mI) was evaluated 40 pre-HD patients [15,16] with an increase in 62.5% and no difference in 37.5% of cases; other metabolites studied, including Cho, total choline (tCho), and total creatine (tCr), did not show significant differences in their concentration [9,10,16]. A summary of all the metabolic changes in this group can be found in Figure 4.

**Figure 5:**
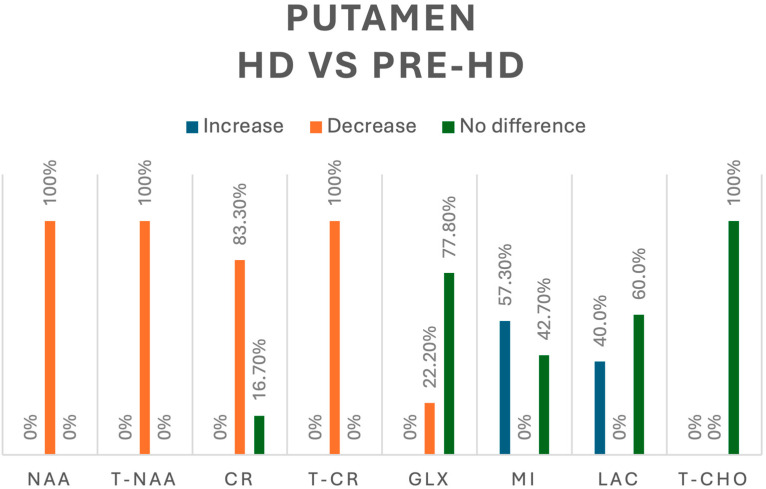
Comparison of metabolites in the putamen between pre-HD and HD patients. N-acetyl-aspartate (NAA), total N-acetyl-aspartate (T-NAA), creatine (CR), total creatine (T-CR), glutamate/glutamine (GLX), myoinositol (MI), lactate (LAC), total choline (T-CHO).

In comparison, when looking into HD vs. pre-HD in the putamen, tNAA (*n* = 64) [8,14,15,16] and NAA (*n* = 41) [10,15] were decreased in 100% of participants, whereas Cr, assessed in 18 HD patients [8,10], was decreased in 83.3% and showed no difference in 16.7% of cases, and tCr was reported as decreased in 29 HD patients [16]. Regarding Glx, 44 HD patients were evaluated; a decreased concentration was reported in 22.2% and no difference in 77.8% [10,14,16]. mI was assessed in 103 HD patients [8,10,14,15,16], and we found an increase in only 57.3% of participants, with no difference in 42.7%. Lactate was assessed in 25 HD patients [10,13,14]; we found an increase in 40% and no difference in 60% of them. Finally, there were no significant differences between pre-HD and HD participants in the concentration of tCho in the putamen [16]. A summary of all the metabolic changes in this group can be found in Figure 5.

#### 3.3.7. Caudate Nucleus

In the caudate, when evaluating pre-HD vs. HC, only two studies analyzed metabolites in this region [5,8]; NAA was assessed in one study, in which all 10 pre-HD patients had a decrease in concentration (*p* < 0.05) [5]. In the same study Cho, Glx, Glu and mI were also assessed, and no differences were detected between HC and pre-HD [5]. The second study assessed tNAA in four pre-HD patients, reporting a significant decrease (*p* < 0.05). Both studies also assessed Cr, and no changes were found between HC and pre-HD patients [5,8].

**Figure 6:**
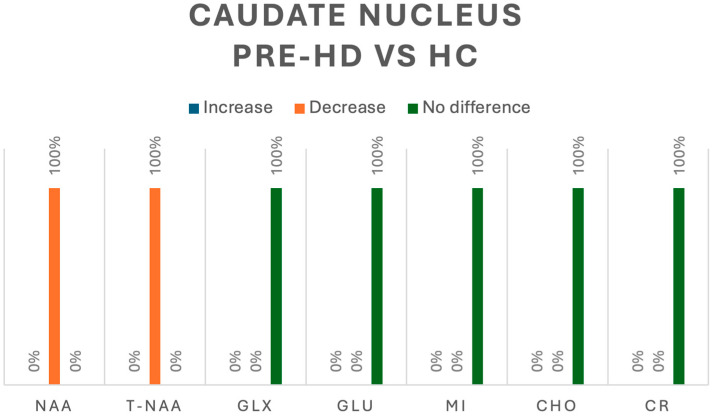
Comparison of metabolites in the caudate nucleus between pre-HD patients and healthy controls (HCs). N-acetyl-aspartate (NAA), total N-acetyl-aspartate (T-NAA), glutamate/glutamine (GLX), glutamate (GLU), myoinositol (MI), choline (CHO), creatine (CR).

**Figure 7:**
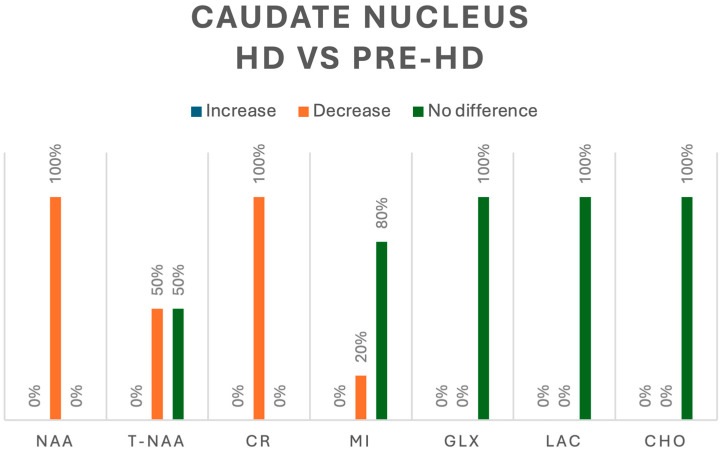
Comparison of metabolites in the caudate nucleus between pre-HD and HD patients. N-acetyl-aspartate (NAA), total N-acetyl-aspartate (T-NAA), creatine (CR), myoinositol (MI), glutamate/glutamine (GLX), lactate (LAC), choline (CHO).

In contrast, when evaluating HD vs. pre-HD, three studies analyzed metabolites in the caudate nucleus [8,10,14]. NAA was evaluated in 12 patients with HD, and a significant decrease was found in 100% [10]; additionally, tNAA was evaluated in 6 patients and only 50% reported a significant decrease [8,14]. Cr was analyzed in 18 patients with HD and 100% showed a significant decrease [8,10], whereas mI was decreased in 20% of 15 patients with HD and showed no difference with that in the remaining 80% in pre-HD patients [10,14]. Finally, Cho, Glx, and Lac, assessed in 15 patients with HD, showed no significant changes in their concentrations when compared with those in pre-HD patients [10,14]. A summary of all the metabolic changes in pre-HD vs. HC and pre-HD vs. HD can be found in Figure 6 and Figure 7, respectively.

## 4. Discussion

To the best of our knowledge, this is the first systematic review that compiles relevant information regarding the use of MRS as a biomarker between HC, pre-HD, and HD; despite the growing utilization of MRS in clinical practise, our review underscores the necessity for additional studies to explore its potential as a biomarker for distinguishing between stages of HD (pre-manifest versus manifest) and as a diagnostic instrument (HC versus HD and HC versus pre-HD). This work seeks to elucidate and, in certain cases, highlight the variability in metabolic alterations between pre-HD and HC, as well as between HD and pre-HD. The identification of these alterations typically aligns with the molecular pathways underlying neurotoxicity and degeneration associated with Huntington’s disease. Our study identifies potential biomarkers that may correlate with serological and structural indicators, which together could be used to evaluate disease progression and enhance our predictive capabilities. Combining MRS with other biomarkers is a promising avenue for enhancing the diagnosis and prognosis of HD. When integrated with genetic biomarkers like the CAG repeat expansion in the HTT gene, MRS significantly improves risk stratification and disease monitoring [1]. Moreover, MRS, when combined with neuroimaging techniques such as MRI and PET, serves as a powerful tool, shedding light on structural and functional brain changes [1,2,3]. However, implementing MRS for early HD poses challenges such as technical variability, limited technological access, and the need for standardized interpretation and execution. Current clinical guidelines focus on genetic testing and neuroimaging, necessitating revisions to include MRS [1,2,3]. Small sample sizes in studies limit generalizability, while cost and patient acceptance also pose barriers, highlighting the need for collaborative efforts to validate MRS as a reliable biomarker for early HD.

In HD, metabolite changes identified through MRS provide important insights into the disease’s pathophysiology. Notably, a relationship exists between these changes and mitochondrial dysfunction, a hallmark of HD; decreased levels of N-acetylaspartate (NAA) and total N-acetylaspartate (tNAA) reflect impaired neuronal integrity and energy metabolism, indicating mitochondrial impairment [17]. Increased lactate levels further suggest a transition to anaerobic metabolism, underscoring the role of mitochondrial dysfunction. Additionally, excitotoxicity is a crucial aspect of HD pathophysiology; elevated choline levels may indicate heightened cell membrane turnover due to excessive glutamate activity, leading to neuronal damage; this excitotoxic process is compounded by elevated levels of glutamate and glutamine, which highlight the detrimental effects of excitatory neurotransmission [18,19]. Furthermore, alterations in myoinositol levels indicate neuroinflammation and gliosis, which are common in HD; elevated myoinositol suggests astrocytic activation and inflammation, contributing to neurodegeneration [20]. Collectively, these metabolite changes offer a comprehensive understanding of the mechanisms underlying HD, linking metabolic dysfunction to neuronal health, excitotoxicity, and inflammatory processes.

We found a statistically significant reduction in NAA and Glu in the frontal cortex, consistent with findings in other previously characterized locations [5]. A decrease in the cross-sectional area of grey and white matter has been documented in the frontal, temporal, insular, and parietal cortical regions in Huntington’s disease grades 2–4 [21]; this atrophy may elucidate the observed reduction in levels of this region. Our research underscores the therapeutic significance of the NAA/Ch ratio, since we observed a statistically significant reduction in the frontal cortex [5]. This reduction signifies neuronal death, as NAA concentrations diminish in advanced stages of Huntington’s disease while staying normal in people with milder clinical impairments. The alignment of the frontal cortex with the degenerative process [6] highlights the potential of the NAA/Ch ratio as a diagnostic instrument. Furthermore, atrophy of a specific region of the frontal cortex, the prefrontal cortex, in HD patients can partly explain the cognitive dysfunction they present [22]. Recent research points to the potential of MRS to detect changes in metabolites such as NAA and Glu in the prefrontal cortex of HD patients, reflecting neuronal loss and impaired excitatory neurotransmission. However, in our study, MRS did not prove to be useful to detect early Huntington disease in the prefrontal cortex, showing no differences in the concentrations of Cr, Cho, NAA, mI, Lac, and Glx [10].

When looking at a different cortical area, we found a statistically significant decrease in tNAA and Cr in the occipital cortex [7]. The concentration of tNAA correlates with neuronal density and neuronal function, whereas creatine is involved in energy metabolism via the creatine kinase reaction, generating ATP [4]. Furthermore, a statistically significant increase in Cho and Lac was found in this region; the elevated choline may reflect gliosis [11]. These findings might indicate that the degenerative process occurring in the striatum (putamen and caudate) might also impact the occipital cortex of HD patients; however, more studies are required to completely elucidate this.

The definitive feature of Huntington’s disease is neuronal degeneration and atrophy of the neostriatum, specifically the putamen and caudate nucleus. Prominent atrophy also occurs in the neocortex, the main input region of the striatum, and in advanced stages of the disease, other brain regions are also affected, as aforementioned [23]. NAA and tNAA were the most extensively researched metabolites in the putamen; a significant reduction in their levels was observed even prior to the manifestation of symptoms [7,8,10,13,14,15,16]. The role of NAA remains inadequately comprehended; yet, it is regarded as a marker of neuronal integrity [8,16]. Moreover, tNAA concentration in grey matter indicates neuronal quantity and health, whereas its concentration in white matter serves as a marker for axonal density [8,16]. Our study revealed a reduction in NAA and tNAA in 100% of HD participants compared to pre-HD individuals, and in 72.5–77.9% of pre-HD participants compared to healthy controls in the putamen (100% for NAA and tNAA in pre-HD versus HC, and 100% for NAA in HD versus HC in the caudate nucleus) [5,7,8,10,13,14,15,16]; this indicates the progressive neuronal degeneration and its significance in disease advancement. Van Oostrom et al. observed no significant disparities in these metabolites between pre-HD and HC, potentially attributable to methodological variations or the extent of biological damage in their pre-HD subjects [9]. NAA and tNAA have demonstrated a correlation with the number of CAG repetitions and with worse scores in motor scales such as the UHDRS [9]. Examining the caudate in further detail, findings about its metabolic alterations have yielded inconsistent outcomes. Particular metabolites, such as NAA, have demonstrated a consistent and statistically significant decline in patients with pre-HD and HD linked to brain injury, as explained before [7]. This link can be attributed to the diminished volume of the caudate nucleus [5]. The data suggest that metabolic alterations in the caudate occurred prior to the distinctive morphological changes. The majority of other metabolites exhibited either inconclusive results or showed no statistically significant alterations. The limited patient sample analyzed for these metabolites in the specific anatomical region, along with the heterogeneity of the studies, may have affected the results, indicating that additional research with a more standardized methodology is necessary to determine if the observed changes are representative of the broader population of HD and pre-HD patients.

The hypothalamus regulates body temperature, hunger, thirst, mood, libido, blood pressure, and sleep. Numerous HD patients report sleep disturbances and metabolic alterations, including weight loss, especially in advanced stages, attributable to neuronal impairment in this brain region [24,25]. Abnormalities in the hypothalamus can be detected using neuroimaging [24] and post-mortem brain dissections [25]. Nonetheless, despite these associations, MRS has not been demonstrated to be effective for the early identification of HD in the hypothalamus. The sole study examining the hypothalamus ascribed this absence of correlation to the interference of cerebrospinal fluid (CSF) in the region of interest (the voxel utilized for measuring metabolite concentration); owing to the atrophy of this area in Huntington’s disease patients, the voxel evaluating the hypothalamus experienced CSF interference, which can distort the measured concentration of metabolites [10]. Finally, the thalamus, which participates in virtually every sensory function and has important connections with the basal ganglia, had similar results to the hypothalamus [9,10]. No difference was detected in NAA, Cho, Cr, Lac, Glx or mI between HC, pre-HD, and HD participants. However, other MRS studies looking into HD and HC (without considering pre-HD) have reported that the N-acetylaspartate + N-acetylaspartylglutamate (NAA + NAAG)/creatine + phosphocreatine (Cr + PCr) ratio was decreased by 9% and the glycerophosphocholine + phosphocholine (GPC + PCh)/Cr + PCr ratio was increased by 17% in HD when compared to those in HC [26]. Furthermore, another study looking at HD versus HC found that the NAA/Cr ratio was decreased in the thalamus of HD participants (*p* = 0.0001) and that a positive correlation existed between the duration of disease and the NAA/Cr ratio [27]. More studies looking into the differences in pre-HD and HD and/or HC focusing in the thalamus and hypothalamus are needed to analyze if these relationships start in the pre-symptomatic period (pre-HD).

In summary, our systematic review revealed significant alterations in metabolites inside the basal ganglia of pre-HD and HD patients. The most extensively examined regions, exhibiting the most pronounced metabolic changes, align with the pathophysiology of Huntington’s disease, particularly affecting the putamen, caudate, and striatum nuclei. While the outcomes for several metabolites are varied and may aid in the diagnosis of Huntington’s disease (HD), N-acetylaspartate (NAA) and total N-acetylaspartate (tNAA) are the most extensively researched and dependable indicators for enhancing accuracy in distinguishing between individuals with HD, pre-HD, and healthy controls (HCs). Variations in NAA concentrations may also correlate with clinical progression and trinucleotide repeat expansion in this condition. A notable reduction in Cr was seen in the comprehensive examination of the striatum. In contrast to the other metabolites, mI and Lac exhibited an increasing pattern in the caudate nucleus and putamen. Nonetheless, more studies, with less heterogeneity and bias, are required to fully recommend the use of MRS as a diagnostic and prognostic tool.

### Limitations

The heterogeneity and small sample sizes exert a considerable influence on our research findings. Variability in methodologies, including discrepancies in magnetic field strengths, sequences, and participant characteristics, complicates the comparison and synthesis of results, which can lead to inconsistent conclusions regarding the efficacy of MRS. However, we tried to mitigate this by showcasing specific information of the MRS techniques used to obtain the presented metabolic changes by including exhaustive data tables that specify TE, TR, field strength, and sequence; this will enable the reader to properly assess the results of each study. Another limitation of our study is the small sample sizes of the included articles; this reduces statistical power, thereby increasing the likelihood of type I and type II errors, undermining the findings’ reliability and generalizability. These issues underscore the critical need for larger, more standardized MRS studies to enhance its robustness and applicability in clinical contexts.

## 5. Conclusions

Our review has highlighted pertinent metabolites and the variations in their concentrations between pre-HD and HC or HD subjects. Our findings might diverge from those in the existing literature on MRS and HD, as the majority of research in the MRS domain overlook pre-HD individuals, concentrating solely on HD versus HC comparisons. We assert that additional MRS studies concentrating on the pre-HD population are essential, as MRS may detect this group and facilitate treatment prior to the onset of severe HD symptoms. We demonstrated that several metabolites exhibit significant differences between pre-HD and HC, as well as between pre-HD and HD patients, across many brain regions; however, we also highlighted the considerable heterogeneity and small sample size in these data. Despite these constraints, our study underscores a significant avenue of research for HD prediction and prognosis. Future research on MRS for the early diagnosis of HD should prioritize standardizing protocols to ensure consistency in magnetic field strength and metabolite quantification; longitudinal studies are essential for tracking metabolic changes over time, while diverse patient cohorts will help assess the impact of genetic and environmental factors. Integrating MRS with genetic testing and neuroimaging can create a more comprehensive diagnostic approach. Finally, developing clinical guidelines for MRS use across different stages of HD (including pre-HD patients) will enhance interpretation and inform treatment strategies.

## Figures and Tables

**Table 1:** Bias assessment of the included studies.

Study	Year	Study Design	Risk of Bias
Padowski et al. [5]	2014	Cross-sectional	Moderately low
Harms et al. [6]	1997	Cross-sectional	Moderately low
Alcauter-Solórzano et al. [7]	2010	Cross-sectional	High
Sturrock et al. [8]	2010	Cross-sectional	Moderately low
Van Oostrom et al. [9]	2007	Cross-sectional	High
Van den Bogaard et al. [10]	2011	Cross-sectional	Moderately low
Jenkins et al. [11]	1998	Cross-sectional	Moderately low
Sánchez-Pernaute et al. [12]	1999	Cross-sectional	High
Reynolds et al. [13]	2005	Cross-sectional	High
Van den Bogaard et al. [14]	2014	Cohort	Moderately low
Sturrock et al. [15]	2015	Cross-sectional	Moderately low
Lowe et al. [16]	2022	Cohort	Moderately low

**Table 2:** Comprehensive metabolic profiles obtained from research studies that incorporated glutamine/glutamate (Glx).

Author (Year)	Model	Magnetic Field Strength	TE	TR	Voxel Size	Sequence	HC (*n*)	Pre-HD (*n*)	HD (*n*)	Anatomical Area	Reported Difference
M. Padowski et al. (2014) [5]	Philips	3 T	3.20 ms	7 ms	1 × 1 × 1 mm	MPRAGE	10	10	-	Caudate Nucleus	NO DIFFERENCE: PRE-HD vs. HC
										Frontal Cortex	NO DIFFERENCE: PRE-HD vs. HC
Van den Bogaard et al. (2011) [10]	Philips 7-Tesla Achieva	7 T	19 ms	2000 ms	0.44 × 0.44 × 0.84 mm	STEAM	18	14	12	Hypothalamus	NO DIFFERENCE: HD vs. PRE-HD
										Thalamus	NO DIFFERENCE: HD vs. PRE-HD
										Caudate	NO DIFFERENCE: HD vs. PRE-HD
										Putamen	**DECREASE**: HD vs. PRE-HD (*p* < 0.05)
										Prefrontal	NO DIFFERENCE: HD vs. PRE-HD
Reynolds et al. (2005) [13]	General Electric, Signa	0.5 T	17 ms	3500 ms	0.56 cm^3^	PRESS PULSE SEQUENCE	10	17	10	Putamen	**INCREASE**: PRE-HD vs. HC (*p* < 0.05)
Van den Bogaard et al. (2014) [14]	Philips	7 T	5.4 ms	11 ms	1.00 × 1.01 × 1.00 mm	GRADIENT ECHO	-	10	3	Caudate Nucleus	NO DIFFERENCE: HD and PRE-HD
										Putamen	NO DIFFERENCE: HD and PRE-HD
										Frontal Cortex	NO DIFFERENCE: HD and PRE-HD
Lowe et al. (2022) [16]	Siemens Prisma	3 T	30 ms	2000 ms	35 × 10 × 15 mm^3^	PRESS	15	15	29	Putamen	NO DIFFERENCE: PRE-HD vs. HC NO DIFFERENCE: HD vs. PRE-HD

**Table 3:** Comprehensive metabolic profiles obtained from research studies that incorporated lactate (Lac).

Author (Year)	Model	Magnetic Field Strength	TE	TR	Voxel Size	Sequence	HC (*n*)	Pre-HD (*n*)	HD (*n*)	Anatomical Area	Reported Difference
Jenkins et al. (1998) [11]	General Electric	1.5 T	272 ms	2000 ms	2–4 mL	PRESS/STEAM	17	8	31	Striatum	**INCREASE**: PRE-HD vs. CONTROL (*p* < 0.01)
										Occipital Cortex	**INCREASE**: HD vs. CONTROL (*p* < 10^−11^)
Van den Bogaard et al. (2011) [10]	Philips 7-Tesla Achieva	7 T	19 ms	2000 ms	0.44 × 0.44 × 0.84 mm	STEAM	18	14	12	Hypothalamus	NO DIFFERENCE: HD vs. PRE-HD
										Thalamus	NO DIFFERENCE: HD vs. PRE-HD
										Caudate	NO DIFFERENCE: HD vs. PRE-HD
										Putamen	NO DIFFERENCE: HD vs. PRE-HD
										Prefrontal	NO DIFFERENCE: HD vs. PRE-HD
Reynolds et al. (2005) [13]	General Electric, Signa	0.5 T	17 ms	3500 ms	0.56 cm^3^	PRESS PULSE	10	17	10	Putamen	**INCREASE**: HD vs. PRE-HD (*p* not reported)
Van den Bogaard et al. (2014) [14]	Philips	7 T	5.4 ms	11 ms	1.00 × 1.01 × 1.00 mm	GRADIENT ECHO	-	10	3	Caudate Nucleus	NO DIFFERENCE: HD and PRE-HD
										Putamen	NO DIFFERENCE: HD and PRE-HD
										Frontal Cortex	NO DIFFERENCE: HD and PRE-HD

**Table 4:** Comprehensive metabolic profiles obtained from research studies that incorporated Naa/Cho ratio, Cho/Cr ratio, and Glu (glutamate).

Author (Year)	Model	Magnetic Field Strength	TE	TR	Voxel Size	Sequence	HC (*n*)	Pre-HD (*n*)	HD (*n*)	Anatomical Area	Reported Difference
L. Harms et al. (1997) [6]	Gyroscan S15 (Philips)	1.5 T	30.90 ms	2000 ms	20 × 20 × 40 mm	SPARS	-	4	17	Frontal Cortex	**NAA/Cho DECREASE**: PRE-HD vs. HD (*p* < 0.05)
											Cho/Cr NO DIFFERENCE: PRE-HD vs. HD
M. Padowski et al. (2014) [5]	Philips	3 T	3.20 ms	7 ms	1 × 1 × 1 mm	MPRAGE	10	10	-	Caudate Nucleus	Glu NO DIFFERENCE: PRE-HD vs. HC
										Frontal Cortex	**Glu DECREASE**: PRE-HD vs. HC (*p* = 0.013)

**Table 5:** Comprehensive metabolic profiles obtained from research studies that incorporated Myoinositol (mI).

Author (Year)	Model	Magnetic Field Strength	TE	TR	Voxel Size	Sequence	HC (*n*)	Pre-HD (*n*)	HD (*n*)	Anatomical Area	Reported Difference
M. Padowski et al. (2014) [5]	Philips	3 T	3.20 ms	7 ms	1 × 1 × 1 mm	MPRAGE	10	10	-	Caudate Nucleus	NO DIFFERENCE: PRE-HD vs. HC
										Frontal Cortex	NO DIFFERENCE: PRE-HD vs. HC
Sturrock A et al. (2010) [8]	Philips	3 T	35 ms	2000 ms	35 mm × 10 mm × 15 mm	PRESS	30	25	30	Putamen	**INCREASE**: HD vs. PRE-HD (*p* < 0.05) **INCREASE**: PRE-HD vs. HC (*p* < 0.05)
Van den Bogaard et al. (2011) [10]	Philips 7-Tesla Achieva	7 T	19 ms	2000 ms	0.44 × 0.44 × 0.84 mm	STEAM	18	14	12	Hypothalamus	NO DIFFERENCE: HD vs. PRE-HD
										Thalamus	NO DIFFERENCE: HD vs. PRE-HD
										Caudate Nucleus	NO DIFFERENCE: HD vs. PRE-HD
										Putamen	NO DIFFERENCE: HD vs. PRE-HD
										Prefrontal Cortex	NO DIFFERENCE: HD vs. PRE-HD
Van den Bogaard et al. (2014) [14]	Philips	7 T	5.4 ms	11 ms	1.00 × 1.01 × 1.00 mm	GRADIENT ECHO	-	10	3	Caudate Nucleus	**DECREASE**: PRE-HD and HD after 2-year follow-up (*p* = 0.015)
										Putamen	NO DIFFERENCE: HD and PRE-HD
										Frontal Cortex	NO DIFFERENCE: HD and PRE-HD
Sturrock et al. (2015) [15]	Philips Achieva MR	3 T	35 ms	2000 ms	35 mm × 31 mm × 31.5 mm	PRESS	30	25	29	Left Putamen	**INCREASE**: HD vs. PRE-HD (*p* < 0.05) **INCREASE**: PRE-HD vs. HC (*p* < 0.05)
Lowe et al. (2022) [16]	Siemens Prisma	3 T	30 ms	2000 ms	35 mm × 10 mm × 15 mm	PRESS	15	15	29	Putamen	NO DIFFERENCE: PRE-HD vs. HC NO DIFFERENCE: HD vs. PRE-HD

**Table 6:** Comprehensive metabolic profiles obtained from research studies that incorporated N-acetyl-aspartate (NAA).

Author (Year)	Model	Magnetic Field Strength	TE	TR	Voxel Size	Sequence	HC (*n*)	Pre-HD (*n*)	HD (*n*)	Anatomical Area	Reported Difference
M. Padowski et al. (2014) [5]	Philips	3 T	3.20 ms	7 ms	1 × 1 × 1 mm	MPRAGE	10	10	-	Caudate Nucleus	**DECREASE**: PRE-HD vs. HC (*p* = 0.023)
										Frontal Cortex	**DECREASE**: PRE-HD vs. HC (*p* = 0.005)
Sturrock A. et al. (2010) [15]	Philips	3 T	35 ms	2000 ms	35 mm × 10 mm × 15 mm	PRESS	30	25	30	Putamen	**DECREASE**: PRE-HD vs. HC (*p* < 0.05)
Van Oostrom et al. (2007) [9]	SIEMENS AG	1.5 T	135 ms	1500 ms	2000 mm^3^	PRESS	8	19		Putamen	NO DIFFERENCE: PRE-HD vs. HC
										Thalamus	NO DIFFERENCE: PRE-HD vs. HC
Van den Bogaard et al. (2011) [10]	Philips 7-Tesla Achieva	7 T	19 ms	2000 ms	0.44 × 0.44 × 0.84 mm	STEAM	18	14	12	Hipothalamus	NO DIFFERENCE: HD vs. PRE-HD
										Thalamus	NO DIFFERENCE: HD vs. PRE-HD
										Caudate	**DECREASE**: HD vs. PRE-HD (*p* < 0.05)
										Putamen	**DECREASE**: HD vs. PRE-HD (*p* < 0.05)
										Prefrontal	NO DIFFERENCE: HD vs. PRE-HD
Jenkins et al. (1998) [11]	General Electric	1.5 T	272 ms	2000 ms	2–4 mL	PRESS/STEAM	17	8	31	Striatum	NO DIFFERENCE: PRE-HD vs. HC
										Occipital Cortex	NO DIFFERENCE: HD vs. CONTROL
Sánchez-Pernaute et al. (1999) [12]	General Electric, Signa Advantage	1.5 T	288 ms	1500 ms	Mean: 4.54 cm^3^ HD: 3.27 cm^3^	PROBE	5	4	6	Striatum	**DECREASE**: PRE-HD vs. HC (*p* < 0.01) **DECREASE**: HD vs. PRE-HD (*p* < 0.01)
Reynolds N. C. et al. (2005) [13]	General Electric, Signa	0.5 T	17 ms	3500 ms	0.56 cm^3^	PRESS	10	17	10	Putamen	**DECREASE**: PRE-HD vs. HC (*p* < 0.05)
Sturrock et al. (2015) [15]	Philips Achieva MR	3 T	35 ms	2000 ms	35 mm × 31 mm × 31.5 mm	PRESS	30	25	29	Left Putamen	**DECREASE**: HD vs. PRE-HD (*p* < 0.001) **DECREASE**: PRE-HD vs. HC (*p* < 0.001)

**Table 7:** Comprehensive metabolic profiles obtained from research studies that incorporated total N-acetyl-aspartate (tNAA).

Author (Year)	Model	Magnetic Field Strength	TE	TR	Voxel Size	Sequence	HC (*n*)	Pre-HD (*n*)	HD (*n*)	Anatomical Area	Reported Difference
Alcauter-Solórzano S et al. (2010) [7]	Excite II, GE	3 T	144 ms	1500 ms	Not reported	PRESS	3	4	3	Caudate Head	**DECREASE**: HD vs. PRE-HD (*p* < 0.05) NO DIFFERENCE: PRE-HD vs. HC
										Putamen	**DECREASE**: HD vs. PRE-HD (*p* < 0.05) NO DIFFERENCE: PRE-HD vs. HC
										Occipital Cortex	**DECREASE**: HD vs. PRE-HD (*p* < 0.05) NO DIFFERENCE: PRE-HD vs. HC
Sturrock A et al. (2010) [8]	Philips	3 T	35 ms	2000 ms	35 mm × 10 mm × 15 mm	PRESS	30	25	30	Putamen	**DECREASE**: PRE-HD vs. CONTROL (*p* < 0.001)
Van den Bogaard et al. (2014) [14]	Philips	7 T	5.4 ms	11 ms	1.00 × 1.01 × 1.00 mm	GRADIENT ECHO		10	3	Caudate Nucleus	NO DIFFERENCE HD and PRE-HD
										Putamen	**DECREASE**: HD and PRE-HD at two-year follow-up (*p* = 0.028)
										Frontal Cortex	NO DIFFERENCE: HD and PRE-HD
Sturrock et al. (2015) [15]	Philips Achieva MR	3 T	35 ms	2000 ms	35 mm × 31 mm × 31.5 mm	PRESS	30	25	29	Left Putamen	**DECREASE**: HD vs. PRE-HD (*p* < 0.01) **DECREASE**: PRE-HD vs. HC (*p* < 0.001)
Lowe et al. (2022) [16]	Siemens Prisma	3 T	30 ms	2000 ms	35 × 10 × 15 mm^3^	PRESS	15	15	29	Putamen	NO DIFFERENCE: PRE-HD vs. HC **DECREASE**: HD vs. PRE-HD (*p* = 0.03)

**Table 8:** Comprehensive metabolic profiles obtained from research studies that incorporated choline (Cho).

Author (Year)	Model	Magnetic Field Strength	TE	TR	Voxel Size	Sequence	HC (*n*)	Pre-HD (*n*)	HD (*n*)	Anatomical Area	Reported Difference
M. Padowski et al. (2014) [5]	Philips	3 T	3.20 ms	7 ms	1 × 1 × 1 mm	MPRAGE	10	10	-	Caudate Nucleus	NO DIFFERENCE: PRE-HD vs. HC
										Frontal Cortex	**DECREASE**: PRE-HD vs. HC (*p* = 0.033)
Van Oostrom et al. (2007) [9]	SIEMENS AG	1.5 T	135 ms	1500 ms	2000 mm^3^	PRESS	8	19	-	Putamen	NO DIFFERENCE: PRE-HD vs. HC
										Thalamus	NO DIFFERENCE: PRE-HD vs. HC
Van den Bogaard et al. (2011) [10]	Philips 7-Tesla Achieva	7 T	19 ms	2000 ms	0.44 × 0.44 × 0.84 mm	STEAM	18	14	12	Hipothalamus	NO DIFFERENCE: HD vs. PRE-HD
										Thalamus	NO DIFFERENCE: HD vs. PRE-HD
										Caudate	NO DIFFERENCE: HD vs. PRE-HD
										Putamen	NO DIFFERENCE: HD vs. PRE-HD
										Prefrontal	NO DIFFERENCE: HD vs. PRE-HD
Jenkins et al. (1998) [11]	General Electric	1.5 T	272 ms	2000 ms	2–4 mL	PRESS/STEAM	17	8	31	Striatum	**INCREASE**: PRE-HD vs. CONTROL (*p* < 0.001)
										Occipital Cortex	**INCREASE**: HD vs. CONTROL (*p* < 0.01 or *p* < 0.02 if Choline/Cr ratios are used).
Sánchez-Pernaute et al. (1999) [12]	General Electric, Signa Advantage	1.5 T	288 ms	1500 ms	Mean: 4.54 cm^3^ HD: 3.27 cm^3^	PROBE	5	4	6	Striatum	NO DIFFERENCE: PRE-HD vs. HC NO DIFFERENCE: HD vs. PRE-HD **DECREASE**: HD vs. HC (*p* < 0.05)

**Table 9:** Comprehensive metabolic profiles obtained from research studies that incorporated creatine (Cr).

Author (Year)	Model	Magnetic Field Strength	TE	TR	Voxel Size	Sequence	HC (*n*)	Pre-HD (*n*)	HD (*n*)	Anatomical Area	Reported Difference
M. Padowski et al. (2014) [5]	Philips	3 T	3.20 ms	7 ms	1 × 1 × 1 mm	MPRAGE	10	10	-	Caudate Nucleus	NO DIFFERENCE: PRE-HD vs. HC
										Frontal Cortex	NO DIFFERENCE: PRE-HD vs. HC
Alcauter-Solórzano S et al. (2010) [7]	Excite II, GE	3 T	144 ms	1500 ms	Not reported	PRESS	3	4	3	Caudate Head	**DECREASE**: HD vs. PRE-HD (*p* = 0.05) NO DIFFERENCE: PRE-HD vs. HC
										Putamen	**DECREASE**: HD vs. PRE-HD (*p* < 0.05) NO DIFFERENCE: PRE-HD vs. HC
										Occipital Cortex	**DECREASE**: HD vs. PRE-HD (*p* < 0.05) NO DIFFERENCE: PRE-HD vs. HC
Van Oostrom et al. (2007) [9]	SIEMENS AG	1.5 T	135 ms	1500 ms	2000 mm^3^	PRESS	8	19		Putamen	NO DIFFERENCE: PRE-HD vs. HC
										Thalamus	NO DIFFERENCE: PRE-HD vs. HC
Van den Bogaard et al. (2011) [10]	Philips 7-Tesla Achieva	7 T	19 ms	2000 ms	0.44 × 0.44 × 0.84 mm	STEAM	18	14	12	Hipothalamus	NO DIFFERENCE: HD vs. PRE-HD
										Thalamus	NO DIFFERENCE: HD vs. PRE-HD
										Caudate	**DECREASE**: HD vs. PRE-HD (*p* < 0.05)
										Putamen	**DECREASE**: HD vs. PRE-HD (*p* < 0.05)
										Prefrontal	NO DIFFERENCE: HD vs. PRE-HD
Sánchez-Pernaute et al. (1999) [12]	General Electric, Signa Advantage	1.5 T	288 ms	1500 ms	Mean: 4.54 cm^3^ HD: 3.27 cm^3^	PROBE	5	4	6	Striatum	**DECREASE**: PRE-HD vs. HC (*p* < 0.05) **DECREASE**: HD vs. PRE-HD (*p* < 0.05) **DECREASE**: HD vs. HC (*p* < 0.001)
Reynolds, N. C et al. (2005) [13]	General Electric, Signa	0.5 T	17 ms	3500 ms	0.56 cm^3^	PRESS	10	17	10	Putamen	NO DIFFERENCE: HD vs. HC **DECREASE**: PRE-HD vs. HC (*p* < 0.05)

## Data Availability

The original contributions presented in the study are included in the article/Supplementary Material, further inquiries can be directed to the corresponding author/s.

## Supplementary Materials

The following supporting information can be downloaded at https://www.mdpi.com/article/10.3390/jcm13216390/s1: File S1: Search Strategy “Psychiatric Disorders and Substance Abuse: A Systematic Review of Neuroimaging Findings”.
