# Supplementary material for: Early Diagnosis of Huntington Disease: Insights from Magnetic Resonance Spectroscopy—A Systematic Review
Source: J Clin Med. 2024 Oct 25;13(21):6390. doi: 10.3390/jcm13216390 (PMC11546511; doi:10.3390/jcm13216390)
Supplement: Supplementary file 1 [file jcm-13-06390-s001.zip › jcm-3249204-supplementary.pdf]

Supplementary Material File S1 (S1)

Search Strategy “Psychiatric Disorders and Substance Abuse: A Systematic Review of Neuroimaging Findings”

PubMed (MEDLINE):

((HUNTINGTON'S DISEASE) OR (HUNTINGTON'S CHOREA) OR (HUNTINGTON'S DEMENTIA) OR (CHRONIC PROGRESSIVE HEREDITARY CHOREA) OR ("HUNTINGTON DISEASE"[MESH])) AND ((NUCLEAR MAGNETIC RESONANCE) OR (MAGNETIC RESONANCE SPECTROSCOPIES) OR (MR SPECTROSCOPY) OR ("MAGNETIC RESONANCE SPECTROSCOPY"[MESH]))

Virtual Health Library (VHL):

((HUNTINGTON'S DISEASE) OR (ENFERMEDAD DE HUNTINGTON) OR (HUNTINGTON’S CHOREA) OR (COREA DE HUNTINGTON) OR (HUNTINGTON’S DEMENTIA) OR (DEMENCIA DE HUNTINGTON) OR (CHRONIC PROGRESSIVE HEREDITARY CHOREA) OR (COREA CRÓNICA HEREDITARIA PROGRESIVA)) AND ((NUCLEAR MAGNETIC RESONANCE) OR (RESONANCIA MAGNETICA NUCLEAR) OR (MAGNETIC RESONANCE SPECTROSCOPIES) OR (ESPECTROSCOPIAS POR RESONANCIA MAGNÉTICA) OR (MR SPECTROSCOPY) OR (ESPECTROSCOPIA) OR (MAGNETIC RESONANCE SPECTROSCOPY) OR (ESPECTROSCOPIA POR RESONANCIA MAGNETICA))

Scopus:

TITLE-ABS-KEY ((HUNTINGTON AND DISEASE) OR (HUNTINGTON’S AND CHOREA) OR (HUNTINGTON’S AND DEMENTIA) OR (CHRONIC AND PROGRESSIVE AND HEREDITARY AND CHOREA)) AND ((NUCLEAR AND MAGNETIC AND RESONANCE) OR (MAGNETIC AND RESONANCE AND SPECTROSCOPIES) OR (MR AND SPECTROSCOPY) OR (MAGNETIC AND RESONANCE AND SPECTROSCOPY))
